# Therapeutic Targeting of MicroRNAs in the Tumor Microenvironment

**DOI:** 10.3390/ijms22042210

**Published:** 2021-02-23

**Authors:** Rebecca Raue, Ann-Christin Frank, Shahzad Nawaz Syed, Bernhard Brüne

**Affiliations:** 1 Institute of Biochemistry I, Faculty of Medicine, Goethe-University Frankfurt, 60590 Frankfurt, Germany; raue@biochem.uni-frankfurt.de (R.R.); frank@biochem.uni-frankfurt.de (A.-C.F.); 2 Project Group Translational Medicine and Pharmacology TMP, Fraunhofer Institute for Molecular Biology and Applied Ecology, 60596 Frankfurt, Germany; 3 German Cancer Consortium (DKTK), Partner Site Frankfurt, 60590 Frankfurt, Germany; 4 Frankfurt Cancer Institute, Goethe-University Frankfurt, 60596 Frankfurt, Germany

**Keywords:** breast cancer, inflammation, macrophage, microRNA, RNA therapy

## Abstract

The tumor-microenvironment (TME) is an amalgamation of various factors derived from malignant cells and infiltrating host cells, including cells of the immune system. One of the important factors of the TME is microRNAs (miRs) that regulate target gene expression at a post transcriptional level. MiRs have been found to be dysregulated in tumor as well as in stromal cells and they emerged as important regulators of tumorigenesis. In fact, miRs regulate almost all hallmarks of cancer, thus making them attractive tools and targets for novel anti-tumoral treatment strategies. Tumor to stroma cell cross-propagation of miRs to regulate protumoral functions has been a salient feature of the TME. MiRs can either act as tumor suppressors or oncogenes (oncomiRs) and both miR mimics as well as miR inhibitors (antimiRs) have been used in preclinical trials to alter cancer and stromal cell phenotypes. Owing to their cascading ability to regulate upstream target genes and their chemical nature, which allows specific pharmacological targeting, miRs are attractive targets for anti-tumor therapy. In this review, we cover a recent update on our understanding of dysregulated miRs in the TME and provide an overview of how these miRs are involved in current cancer-therapeutic approaches from bench to bedside.

## 1. Introduction

Tumors consist of rapidly dividing neoplastic cells and stroma that comprises connective tissue, blood vessels, and various host cells. The interactions between these cells via various mediators define a particular tumor-microenvironment. Growing tumors reprogram tumor-associated host cells to perform pro-tumoral functions. This is usually achieved by means of various tumor-derived factors that are dynamically interposed between tumor cells and host stroma. The continued relationship of several factors and different types of host with malignant cells makes it difficult to design efficient cancer therapeutic agents. In 2020, Globocan estimated over 19.2 million new cancer cases and 9.96 million deaths worldwide, suggesting that there are still hardships encountered during cancer diagnosis and treatment. Combinatorial therapies, where more than one drug is administered to target different cancerous molecules or pathways, have been shown to yield better therapeutic outcomes. However, they are usually cost-intensive, require complicated treatment, and bear the risk of undesirable drug–drug interactions. Importantly, with the progress in genome sequencing, new classes of non-coding RNAs (ncRNA) have been identified that may open the way for designing gene modulating anti-cancer agents. These ncRNAs include microRNAs (miRs), natural antisense transcripts, piwi-interacting RNAs (piRNAs), and long ncRNAs (lncRNAs), with miRs being the most studied ncRNA candidates in clinical research [1,2,3].

MiRs are small regulatory RNA molecules of around 18–25 nucleotides in length. They bind to the 3′ untranslated region (UTR) of mRNA targets, thereby regulating gene expression. While perfect pairing of the miR with the target mRNA promotes mRNA degradation, imperfect pairing may repress protein translation [4]. MiRs have also been shown to increase target protein levels by either stabilizing mRNA translation [5], or affecting the transcription of target genes in the nucleus [6], but these events seem to occur less frequently. Since the base-pairing does not have to be perfectly complementary to the target sequence, a single miR can regulate the translation of multiple mRNAs [7,8,9,10]. In addition, the 3′ UTR of a single mRNA is frequently targeted by several different miRs, suggesting that miRs cooperate to fine tune gene expression [9,11]. Since miRs orchestrate tissue homeostasis, it is reasonable to think that their impaired expression causes dysregulation in cancerous networks. Considering that miRs regulate several genes and protein networks, restoration of the normal miR programs in cancer and tumor infiltrating host cells might aid in reversing cancer phenotypes [12,13]. Thus, developing miR-based therapies may prove to be more comprehensive and successful than targeting individual proteins or genes.

Since cancer is a complex system of malignant cells and infiltrating host cells, it is reasonable that such anti-tumoral miR therapeutic agents will not only act on malignant cells, but also on other cells of the tumor microenvironment (TME), including tumor-associated macrophages (TAMs) and cancer-associated fibroblasts (CAFs). TAMs are the most abundant immune cell type in the TME and are associated with crucial tumor hallmarks, e.g., angiogenesis, invasion, motility, intravasation, survival, and premetastatic site formation [14]. In response to factors of the TME they can adapt their phenotype towards pro- (M1-like) or anti-inflammatory (M2-like) functions, and subsequently suppress or promote tumor initiation and progression. MiRs engaged in these processes have been reviewed recently [15].

MiRs can also exist in a stable cell-free form in body fluids and other extracellular environments, including plasma, serum, urine, saliva, seminal, ascites, amniotic pleural effusions, and cerebrospinal fluid [16,17], and act as signaling molecules through paracrine and even endocrine signaling [18,19,20]. Thus, miRs closely linked to malignant phenotypes can be utilized for diagnostic, prognostic, and predictive purposes by measuring their amounts noninvasively in human serum or plasma samples, even in the initial stages of cancer [21,22,23,24,25]. Since most screenings fail to identify tumors in their early stages and biopsies and surgeries of solid tumors are often taken when cancer has considerably developed, measuring miR expression in blood samples is an indispensable tool for early cancer diagnosis [21,23]. Moreover, the development of advanced RNA chemistry and strategies to deliver RNA molecules to target tissues in vivo has now enabled miR-based agents to move into clinical trials. In this review, we will discuss the latest discoveries related to miR-based therapeutics in cancer and provide an overview about miR mimics and antagomiRs that are currently in clinical trials and their combination with other anti-cancer agents. Since cancer is a complex system comprising not only malignant cells but also cells of the immune system, we will also shed light on how miR-based anti-cancer agents might impact on the TME.

## 2. Mechanisms of MiR Dysregulation in the TME

Over the past decade it has become clear that miRs are differentially expressed in many human malignancies [11,26] and associated with stage, progression, and metastasis of cancer [27]. Understanding the underlying mechanisms of miR dysregulation in malignant cells, that include chromosomal aberrations, changes in transcriptional control, epigenetic changes, and defects in the miR biogenesis pathway, is important to develop therapeutical anti-cancer strategies [28]. In this section, we summarize mechanisms that alter the miR profile of malignant cells. We also show some examples of how the TME can alter the miR profile of tumor-infiltrating TAMs, CAFs, and other host cells, which has been reviewed recently [29,30].

### 2.1. Mechanisms of MiR Dysregulation in Malignant Cells

In malignant cells of hematopoietic and solid tumors, a plethora of non-random chromosomal abnormalities have been discovered, and miR genes frequently reside in such cancer-associated genomic regions [31]. These regions may be tumor suppressor gene-containing minimal regions showing a loss of heterozygosity, or oncogenes harboring minimal regions of amplification, or fragile sites or general breakpoint regions of the genome [31]. In B-cell chronic lymphocytic leukemia, the miR-15a/16-1 cluster gene at chromosome 13q14 is lost [32] whereas in lung cancer, the decreased expression of miR-143 and miR-145 is due to the loss of the 5q33 region harboring both miR genes [33]. On the other hand, the miR-17-92 cluster, which comprises seven miRs and resides in intron 3 of the C13orf25 gene at 13q31.3, is frequently amplified in B-cell lymphomas [34], lung cancer [35], and T-cell acute lymphoblastic leukemia [36], resulting in overexpression of these miRs. Furthermore, Genomic alterations in miR loci have been shown in melanoma, ovarian and breast cancer [37]. These findings implicate that the aberrant expression of miRs in malignant cells as compared to healthy tissue can often be attributed to amplification, deletion, or translocation of miR genes and miR gene locations.

In addition, the expression of miRs is tightly controlled by various transcription factors that induce the transcription of precursor-miRs, thereby increasing the miR expression. In cancer, deregulation of key transcription factors, such as c-Myc and p53, results in the dysregulated expression of miRs and subsequently promotes tumor development. c-Myc is frequently upregulated in different types of cancer, where it regulates cell proliferation and apoptosis, and transactivates the expression of the oncogenic miR-17-92 cluster by binding to E-box elements in the miR-17-92 promoter [38,39]. Chang et al. analyzed human and mouse models of B cell lymphoma and demonstrated that consistent with its role as an oncogene, c-Myc represses the transcriptional activity of tumor suppressive miRs such as miR-15a, miR-26, miR-29, miR-30, and let-7 families [40]. In hepatocellular carcinoma (HCC), c-Myc represses miR-122 expression that in turn indirectly inhibits c-Myc transcription by targeting the transcription factor Dp-2 (Tfdp2) and E2F transcription factor 1 (E2f1), which are essential for tumor development [41]. Another example of how a transcriptional factor can control miR expression is the miR-34-p53 axis, which mediates tumor suppressive functions [42]. p53 is a tumor suppressor and regulates the expression of many genes, thereby controlling cell cycle progression, apoptosis, and senescence. p53 is activated upon DNA damage and oncogenic stress and induces the expression of miR-34a to trigger apoptosis. In turn, miR-34a downregulates sirtuin 1 (SIRT), a negative regulator of p53 by deacetylation, causing a positive feedback loop [43,44,45]. p53 also controls the expression of several other miRs, such as miR-605 [46], miR-1246 [47], miR-107 [48], and miR-145 [49]. Huang et al. found that upon hypoxia, the transcription factor hypoxia-inducible factor-alpha (HIF1α) regulates the expression of miR-210 in multiple tumor types through a hypoxia-responsive element (HRE) [50]. Similarly, miR-155 also contains a functional HRE in its promoter and is induced by HIF1α in epithelial cells [51], where it contributes to the resolution of HIF effects upon chronic hypoxia by directly inhibiting HIF mRNA. The miR-200 family genes have been shown to be tightly regulated by the zinc-finger E-box-binding homeobox transcription factors ZEB1 and ZEB2, which are the key activators of epithelial-to-mesenchymal transition (EMT) [52]. In addition, the ligand activated transcription factors and hormone receptors estrogen receptor (ER) and androgen receptor (AR) can indirectly change miR abundance through several signaling pathways but also activate the transcription of certain miRs in cancer. For instance, miR-515 is transcriptionally repressed by ERα and functions as a tumor suppressor in breast cancer cells by increasing the level of oncogenic sphingosine kinase 1 (SK1) [53]. In breast cancer, ER negatively modulates the miR-221/222 gene by recruitment of transcriptional repression partners leading to enhanced proliferation and migratory activity [54]. Like estrogen/ER, androgen/AR can directly regulate oncomiRs, such as miR-125b, miR-21, miR-221/222, miR-27a, and miR-32 [55] and tumor-suppressor miRs, miR-135a, and miR-141 [56,57], which has been validated by chromatin immunoprecipitation analysis.

In addition, miRs, similar to protein-coding genes, are susceptible to epigenetic modulations, which include DNA hypomethylation, hypermethylation of CpG islands in tumor suppressor genes, and disruption of histone modification patterns [58,59]. For instance, in acute myeloid leukemia (AML) the acute myeloid leukemia-associated fusion protein AML1/ETO induces the heterochromatic silencing of miR-193a through CpG methylation, thereby contributing to leukemogenesis [60]. In T24 bladder cancer cells, treatment with DNA methylation and histone acetylation inhibitors results in more than 3-fold upregulation of 17 of 313 human miRs [61]. Interestingly, miR-127, which is embedded in a CpG island and frequently downregulated in cancer cells, was upregulated upon treatment resulting in downregulation of the protooncogene B-cell lymphoma 6 (BCL6), indicating that DNA demethylation and histone deacetylase inhibition can activate the expression of miRs that may act as tumor suppressors. Lujambio et al. treated lymph node metastatic cancer cells with a DNA demethylating agent, resulting in hypermethylation-associated silencing of miR-148a, miR-34b/c, and miR-9 [62]. Restoring those miRs inhibited motility and reduced tumor growth and metastasis formation in vivo. Similarly, miR-9-1, miR-124a, and miR-145-5p are epigenetically silenced by DNA hypermethylation in breast, lung, and colon carcinoma, respectively [63,64,65], which highlights the crucial role of epigenetic regulations in miR expression during tumorigenesis.

The maturation of miRs from primary miR precursors is tightly controlled by a few enzymes and regulatory proteins such as Drosha, Dicer, DiGeorge syndrome critical region 8 (DGCR8), Argonaute (AGO) proteins, and exportin-5, suggesting that the mutation or aberrant expression of those components of the miR biogenesis system provokes aberrant miR expression, which is associated with tumor progression. Recent studies showed that the two key RNase III endonucleases Drosha and Dicer, which are responsible for the precursor-miR and miR-miR* duplex formation, are deregulated in various tumors. Thomson et al. demonstrated that a large fraction of miR genes is regulated post-transcriptionally at the Drosha-processing step, downregulating various miRs in cancer and upon embryonic development [66]. Moreover, single-nucleotide substitution/deletion mutations of DGCR8 and Drosha have been found to occur in 15% of 534 Wilms’ tumors, leading to a significantly decreased expression of mature let-7a and the miR-200 family [67]. Iliou et al. reported that the impairment of Dicer1 in colorectal cancer results in the downregulation of miRs, such as miR-34a, miR-126, and miRs of the miR-200 family, resulting in enhanced stemness features and EMT as well as a greater capacity for tumor initiation and metastasis [68].

### 2.2. Mechanisms of MiR Dysregulation in Tumor-Infiltrating Host Cells

The regulation of miR expression in TAMs, CAFs, and other tumor-infiltrating host cells can be mainly ascribed to soluble factors of the TME or the direct transfer and uptake of exogenous miRs [29,30]. miR-155 is one of the crucial miRs that is involved in the pro-inflammatory functions of macrophages and is persistently downregulated in TAMs. He et al. showed that in HCC this downregulation is due to soluble factors secreted by tumor cells, and restoration of miR-155 in TAMs promoted indirect anti-tumor responses by T-cell activation [69]. In plasmacytoid dendritic cells (DCs), miR-155 is induced in later stages of toll-like receptor 7 (TLR7) activation to repress the expression of interferon (IFN)-α/β [70]. In CAFs of gastric cancer, miR-145 is induced by transforming growth factor (TGF)-β, enhancing α-smooth muscle actin (α-SMA) expression [71]. Chromosomal aberrations also control miR expression in cancer associated stromal cells. By using proteomic and expression profiles, Broniszet et al. reported that the ablation of the phosphatase and tensin homolog (PTEN) gene in mammary stromal fibroblasts activates an oncogenic secretome, accompanied by the downregulation of miR-320 and the transition of NFs into CAFs in breast cancer [72].

Tumor cells use their own miR repertoire to hijack tumor-promoting functions of immune cells, including TAMs, which has been reviewed by our group recently [29]. Several mechanisms of miR transfer have been described, for instance the delivery via exosomes, microvesicles, or apoptotic bodies. For instance, Park et al. demonstrated that upon hypoxia, tumor cells secrete exosomes containing let-7a that directly targets the insulin-protein kinase B (AKT)-mammalian target of rapamycin (mTOR) pathway in TAMs and induce the expression of TAM-associated genes [73]. Pancreatic cancer cells deliver microvesicles containing miR-155 to NFs, thereby stimulating their reprogramming towards CAFs [74]. Recently, we showed that apoptotic breast cancer cells release low-density lipoprotein-bound miR-375, which could be taken up by TAMs to induce their infiltration and migration towards tumor sites [75]. However, how the TME can regulate the expression of miRs in different subsets of tumor-infiltrating host cells needs to be further investigated and might provide novel approaches for immunomodulatory-based therapies.

**Figure 1:**
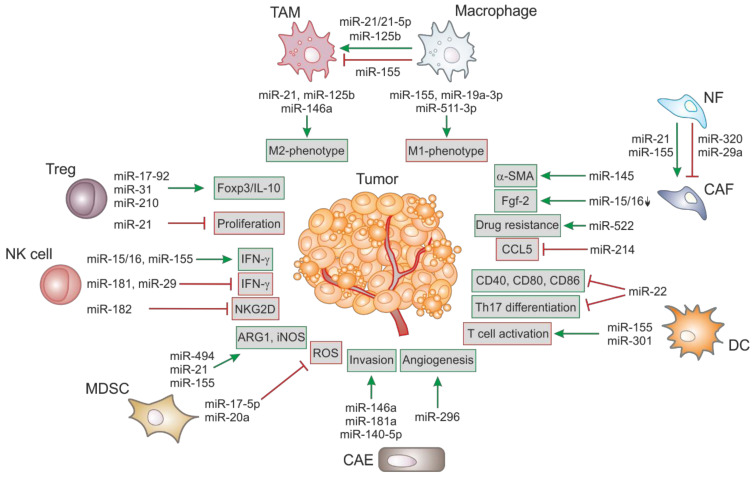
Examples of dysregulated microRNAs (miRs) in cells of the tumor microenvironment (TME) and their impact on tumor cells. MiRs can either act as tumor-suppressors by regulating molecules or pathways with anti-tumoral characteristics (red box; red T bar) or oncomiRs that directly or indirectly impact on tumor-promoting genes and protein networks (green box; green arrow). The differential expression of miRs in macrophages and tumor-associated macrophages (TAMs), or the uptake of exogenous miRs, modulate their polarization. Similarly, miRs expressed in cancer-associated fibroblasts (CAFs) regulate their migration, cytokine production, and trans-differentiation as well as tumor growth. CAF-derived miRs (e.g., miR-522) can also enhance drug resistance of tumor cells. In dendritic cells (DCs), miRs regulate Th17 differentiation, co-stimulatory molecule expression, and T cell activation. miRs expressed in cancer-associated endothelial cells (CAEs) regulate the microvascular invasion and angiogenesis activity to drive tumorigenesis. In myeloid-derived suppressor cell (MDSCs), miRs modulate the expansion/immune-suppressive functions. In natural killer (NK) cells, miRs modulate the production of effector molecules (e.g., IFN-γ) and the activating receptor encoded by killer cell lectin like receptor K1 (NKG2D). Furthermore, miRs regulate the expression of transcription factors and cytokine production of regulatory T cells (Tregs). ARG1, arginase 1; α-SMA, α-smooth muscle actin; CAE, cancer-associated endothelial cell; CAF, cancer-associated fibroblast; DC, dendritic cell; Fgf2, fibroblast growth factor 2; IFN, interferon; iNOS, inducible NO synthase; NF, normal fibroblast; NKG2D, encoded by killer cell lectin like receptor K1; ROS, reactive oxygen species; TAM, tumor-associated macrophage; Treg, regulatory T cell.

MiRs can act as tumor-suppressors by regulating the expression of oncogenes and/or genes that control differentiation and apoptosis and are frequently downregulated upon malignant transformation. On the other hand, several oncogenic miRs are upregulated upon tumorigenesis to enhance tumor development. An overview of deregulated miRs in different stromal cells and their impact on tumorigenesis is shown in Figure 1.

## 3. Therapeutic Modulation of MiRs in Cancer

The current understanding of cancer as a “signaling pathway disease” implies that for successful cancer treatment, therapeutic strategies are required that interfere with multiple oncogenic pathways [76]. Since a single miR can regulate multiple oncogenes or oncogenic pathways that are commonly deregulated in cancer, the therapeutic restoration of tumor suppressor miRs through miR mimics or the suppression of oncomiRs by using antagomiRs provides an appealing tool for cancer therapy.

### 3.1. Restoration of Tumor Suppressive MiRs

MiR mimics are synthetic oligonucleotide duplexes and have the same sequence as the depleted, naturally occurring miR counterpart. Thus, miR mimics were expected to target the same set of mRNAs, suggesting that nonspecific off-target effects are unlikely. They can be chemically modified to have higher stability or to enable the systemic delivery to target cells by using modes and technologies that are also used for siRNAs [77]. Moreover, miR mimics are much smaller than proteins, implying that they can easily enter cells. Besides these advantages of miR mimics as therapeutic molecules the strongest rationale is that a single miR mimic may be designed to target multiple genes and multiple pathways. In 2014, a computational software, miR-Synth, was developed to design miR mimics and the prediction of their mRNA targets and altered signaling pathways [78]. Another strategy to replenish deficient miRs is to transfect cancer cells with adenoviral, lentiviral, or retroviral vectors expressing the tumor-suppressive miR for anti-tumor effects [79]. All these miR replacement strategies seek to activate those cellular programs that are required for cellular homeostasis and interfere with oncogenic signaling cascades that are required for the malignant properties.

To date, various tumor suppressor miRs have been identified in vitro and in vivo and some of them have already been tested in proof-of-concept studies for miR replacement therapy in preclinical animal models. For instance, in non-small-cell lung cancer (NSCLC), exogenous delivery of let-7 to established tumors efficiently restrained tumor growth by blocking cell proliferation and cell cycle pathways [80,81]. Treatment of prostate tumor xenografts with the miR-15a and miR-16-1 mimic caused growth arrest, apoptosis, and inhibited proliferation [82]. Similarly, ectopic expression of the miR-15/16 cluster using viral vectors significantly reduced tumor volume and growth in the MEG01 subcutaneous model of leukemia [83]. In xenograft mouse models of malignant pleural mesothelioma and NSCLC, the tumor-targeted delivery of miR-16 by using a EGFR-targeted EnGeneIC Delivery Vehicle (EDV) and nanocell delivery system (TargomiRs) significantly reduced tumor growth [84]. Another miR that has been demonstrated to be a potential anticancer therapeutic in several studies in vivo is the master tumor-suppressor miR-34. For instance, intra-tumoral and intravenous administration of lipid nanoparticle-encapsulated miR-34 mimics markedly inhibited tumor growth in mouse models of lung, liver, and prostate cancer [85,86,87]. In a *Kras;Trp53* NSCLC mouse model, co-delivery of let-7 and miR-34 by using the same lipid nanoparticle carrier resulted in a significantly reduced tumor burden [88]. Due to their strong anti-tumor effects, lipid nanoparticle-encapsulated miR-34 mimics were tested in a phase I clinical trial (NCT01829971) in several solid and hematological malignancies. Moreover, delivery of members of the miR-200 family using 1,2 dioleoyl-sn glycero-3 phosphatidylcholine (DOPC)-lipid nanoparticles in orthotopic mouse models of ovarian (miR-200a/b), basal-like breast (miR-141), and lung (miR-200a/b) cancers was shown to significantly reduce tumor nodules and metastasis [89]. In a parallel study, Cortez et al. demonstrated that miR-200c upregulation increases intracellular reactive oxygen species by regulating the oxidative stress response genes peroxiredoxin 2 (PRDX2,) NF E2 related factor 2 (NRF2), and sestrin 1 (SESN1) [90]. The systemic delivery of miR-200c in a xenograft lung cancer model fosters tumor cell apoptosis and increased radiosensitivity. miR-mimics have also been used in preclinical trials to induce repolarization of TAMs. In a mouse model of lung cancer, the combinatorial delivery of the pro-inflammatory miR-125b mimic together with wt-p53 cells using CD44/epidermal growth factor receptor (EGFR)-targeted hyaluronic acid-based nanoparticles repolarized TAMs towards the M1 phenotype and inhibited tumor growth [91]. Similarly, the targeted delivery of miR-99b in HCC or subcutaneous Lewis lung cancer mice re-educated TAMs from M2 to M1 phenotype by targeting κB-Ras2 and/or mTOR, thereby enhancing immune surveillance and impeded tumor growth [92]. In a xenograft mouse model of oral squamous cell carcinoma, overexpression of miR-34a-5p by miR mimic significantly inhibited tumorigenesis [93].

### 3.2. Suppression of OncomiRs

It is well accepted that oncogenic miRs are increased in cancer tissues and inhibit important tumor-suppressor genes, resulting in enhanced cell turnover and cell proliferation. Inhibition of oncogenic miRs has become an important area for gene therapy since the restoration of tumor suppressor genes is the pre-requisite to restore normal cellular homeostasis. Thus, inhibition of oncomiRs represents a useful strategy in the fight against cancer. Several different methods have been established to either prevent the binding of oncomiRs to their targets or interfere with the mRNA targets without affecting the miR activity. Synthetic antisense oligonucleotides (ASOs; anti-miR) are single stranded nucleic acids that are around 20-25 bases long. They are designed to complementarily bind to their mature miRs targets, thereby preventing the interaction of that miR with its mRNA target and the consequent normal translation [94]. ASOs can be structurally or chemically modified to make them more resistance to nuclease-mediated degradation, enhance their penetration across the cell membrane, binding affinity, and thermal and metabolic stability [95,96]. Recently, a comprehensive guide for designing anti-miR oligonucleotides has been reported [97]. The therapeutic potential of ASOs have been shown in different types of cancer in vitro and in vivo. For example, inhibition of the anti-apoptotic miR-21 by anti-miR oligonucleotides activates apoptosis and reduces tumor growth in breast cancer [98]. Griveau et al. showed that miR-21 can be silenced by locked nucleic acid (LNA)-modified oligonucleotides in glioblastoma, resulting in reduced cell viability and enhanced intracellular caspase amounts [99]. There are also some studies showing that ASOs can be used to repolarize TAMs towards a pro-tumoral phenotype, thereby reducing tumor burden. For instance, miR-100 is highly expressed in TAMs and maintains pro-tumoral functions by targeting the mTOR signaling pathway. Intra-tumoral treatment of miR-100 antagomiR together with cisplatin significantly reduced tumor metastasis and the invasion capacity in a 4T1 mouse breast cancer model [100]. MiR-21 has been reported to be involved in the metabolic alteration of CAFs in vitro. Treatment of CAFs with a miR-21 antagomiR upon indirect coculture with the pancreatic cancer cell line BxPc-3 reduced glycolysis and lactate acid production in CAFs and decreased oxidative phosphorylation and invasion of tumor cells [101]. For more target specificity, ASOs can also be coupled to ligands (e.g., to be specifically recognized by receptors on the surface of the blood–brain barrier) or arginine-rich cell penetrating peptides to support receptor-mediated endocytosis and direct membrane translocation, respectively [102].

Another strategy to inhibit oncogenic miRs are miR sponges, which are short transcripts that contain multiple artificial miR binding sites. These binding sites are complementary to the specific miR target or a miR family sharing the same seed region and thus sequester endogenous miR in a sequence specific manner [103]. miR sponges are used in several cancer types such as breast, lung, renal, colorectal, and melanoma to effectively inhibit oncomiRs, including miR-19, miR-155, miR-221, and miR-222 to exhibit anti-cancer effects in vitro and in vivo [104,105]. MiR-9 is upregulated in breast cancer cells and inhibits the expression of the tumor suppressor gene CDH1. Treatment of tumor cells with miR sponges containing several miR-9 binding sites inhibited the oncogenic function of miR-9 and effectively restored CDH1 expression, thereby suppressing metastasis [106]. Interestingly, circular miR sponges have been found to be more effective in inhibiting their miR targets compared to their linear counterparts in malignant melanoma and gastric cancer cells [107]. In the last years, several approaches have been investigated to enhance the cytotoxic effect of chimeric antigen receptor (CAR) T-cell to kill cancer cells and miR sponges might be a helpful tool to optimize CAR expression [108].

Since ASO-based agents prevent the binding of oncogenic miRs to their target mRNAs and reactivate the normal activity of genes that were repressed, they were also described as miR-masking oligonucleotides [109]. The function of miR-masking oligonucleotides was first described in breast cancer, where the inhibition of the tumor suppressor TP63 could be prevented by miR-196a2 masking oligonucleotide, reducing tumor cell proliferation [110]. In glioblastoma cells, the miR-9 masking oligonucleotide prevented the interaction of miR-9 with its target PTCH1 and overcame temozolomide resistance, confirming the therapeutic potential of these RNA agents [111].

In addition, the clustered regularly interspaced short palindromic repeats (CRISPR)-associated nuclease 9 (Cas9) system has been demonstrated to efficiently inhibit the expression of oncogenic miRs, including miR-17, miR-21, miR-141, and miR-3188 to reduce tumor cell proliferation, invasion, but to enhance apoptosis [112,113]. This technology can also be used to introduce mutations in key enzymes of the biosynthesis machinery of specific oncogenic miR precursors to prevent the expression of the mature oncomiR [114]. Moreover, CRISPR/Cas9 enhances the sensitivity to chemotherapeutic agents, including cisplatin and paclitaxel [112]. In both in vitro and in vivo models, downregulation of miRs by the CRISPR/Cas9 system is highly stable and could last for 30 days, making it a promising approach for anti-cancer therapy [115].

In 2017, Patutina et al. first described a novel technique to efficiently inhibit oncomiRs by covalently binding an artificial ribonuclease or catalytic peptide to a miR-targeting oligonucleotide to mediate miR degradation [116]. They investigated several miRNases and one of them specifically reduced miR-21 in lymphosarcoma cells, resulting in restoration of key tumor-suppressor proteins and suppressing tumor cell proliferation without appreciable off target effects.

**Figure 2:**
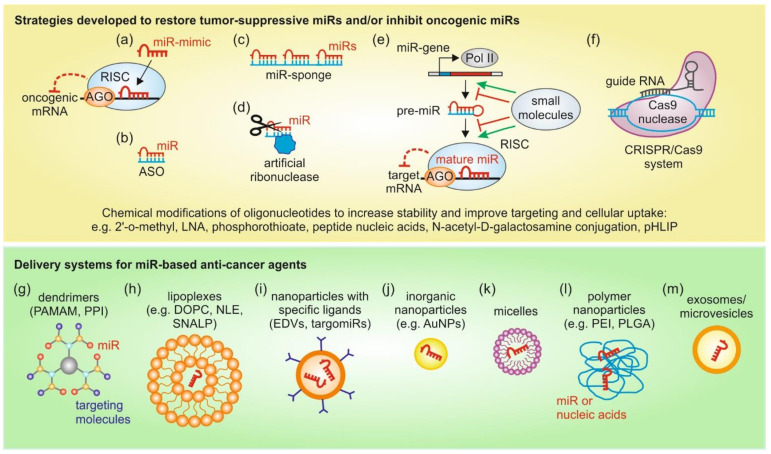
Therapeutic modulation of miR expression and miR carriers. Tumor suppressive miRs can be replenished by miR mimics (**a**), thereby suppressing translation of mRNAs encoding for oncogenes. On the other hand, oncogenic miRs can be inhibited by ASOs (**b**), miR-sponges (**c**), artificial ribonucleases (**d**), small molecules (**e**), or the CRISPR/Cas9 system (**f**). Small molecules have been shown to either suppress oncomiRs or globally enhance miR expression. To increase oligonucleotide stability, chemical modifications can be inserted. Several delivery systems have been established to further increase the stability of the miR therapeutic agent and improve tumor cell targeting, e.g., cationic dendrimers (**g**), lipoplexes (**h**), nanoparticles with tumor-specific ligands (**i**), inorganic nanoparticles (**j**), micelles (**k**), polymer nanoparticles (**l**), and exosomes/microvesicles (**m**). See text for more details. AGO, argonaute protein; ASOs, antisense-oligonucleotides; AuNPs, gold nanoparticles; DOPC, 1,2 dioleoyl-sn glycerol-3 phosphatidylcholine-lipid nanoparticles; EDVs, EnGeneIC Delivery Vehicle; LNA, locked nucleic acid; NLE, neutral lipid emulsions; PAMAM, poly(amidoamine); PEI, poly(ethyleneimine); pHLIP, pH low insertion peptide; PLGA, Poly(lactide-co-glycolide); Pol II, RNA-polymerase II; PPI, poly(propylenimine); RISC, RNA-induced silencing complex.

In essence, several techniques and technologies can be exploited for therapeutic modulation of miRs in a context dependent manner by carefully weighing the pros-and-cons of those approaches. An overview of the above discussed strategies to modulate miR expression and delivery systems for miR therapeutic agents is depicted in Figure 2.

## 4. Combination with Cancer Therapy to Counteract MiR-Mediated Therapy Resistance

Cancer therapy is often associated with several disadvantages, which include toxicity to non-malignant cells or drug resistance. Thus, the demand for new therapy approaches is high and miR therapeutics seem to have high potential, at least as an adjuvant therapy.

Several miRs are involved in chemotherapy resistance and upregulation of oncomiRs is as disruptive for chemotherapy as downregulation of tumor suppressor miRs. For instance, upregulation of miR-155 has been shown to be involved in chemotherapy resistance in several different cancers [117], while for miR-34 its low expression is associated with poor treatment response [118]. For both miRs, and several other prominent ones, their involvement in therapy resistance has been reviewed extensively by others [117,118,119,120,121,122]. However, the knowledge about miR involvement in chemotherapy resistance can be exploited to determine the efficiency of therapy strategies. Several miRs are used as biomarkers to predict clinical outcome and as indicators for therapeutic efficiency of radio-, chemo-, or immunotherapy [123,124,125]. For instance, the oncomiR miR-10b is inhibited by linifanib, which reverses its oncogenic effect. However, at higher expression levels, miR-10b could “hijack” linifanib during cancer treatment and reduce its anti-tumor efficacy by reducing its kinase inhibitory effects. Therefore, miR-10b expression levels may serve as a biomarker to select patients for linifanib treatment [126]. Furthermore, the efficacy of immunotherapy in NSCLC can likely be predicted using miR-320d, miR-320c, and miR-320b as biomarkers [127]. Being able to predict the therapy efficacy before starting the treatment could be valuable in taking treatment decisions and potentially safe lives by providing rationales for applying more promising treatment strategies. Thus, miR-biomarkers predicting the treatment success show a high potential in clinical therapy assignment.

Once patients developed chemotherapy resistance, a promising approach to overcome chemotherapy resistance is the combined delivery of chemotherapeutics and miRs to sensitize tumor cells to chemotherapy, e.g., by targeting of DNA-damage response or cell cycle genes as well as genes related to apoptosis or multidrug resistance by co-delivered miRs [120,128]. However, in this review, we focus on the involvement of TME-derived or induced miRs in immune evasion and therapy resistance as well as on therapeutic candidates that deliver miRs and chemotherapeutics combined in nanoparticles to exploit their synergistic effect.

### 4.1. MiRs as Possible Immunotherapeutics—The Role of MiRs in Immune Evasion in the TME

Immune evasion is one of the emerging hallmarks of cancer and is associated with bad prognosis. TME-mediated miRs have been shown to be one of the regulators involved in immune evasion. As they are affecting all cell types in the TME, there are several points of intervention for combining miR and immunotherapeutic therapies. An important cell type in immune evasion are macrophages as they play a crucial role in activating other immune cells in the TME. For instance, miR-146a-5p neutralization affects the crosstalk between tumor cells and macrophages, thereby changing the entire TME. The underlying mechanism is a miR-146a-5p-induced block of the inducible nitric oxide synthase (iNOS), thereby inhibiting nitric oxide synthesis and subsequently conferring resistance to macrophage-induced cell death in mouse renal carcinoma and colon carcinoma CT26 cell lines. The combination of miR-146a-5p suppression with macrophage therapy could enhance infiltration of cytotoxic CD8^+^ T cells and thus successful anti-tumor immunity in vivo [129]. HCC cells achieve immune evasion by exosome-mediated upregulation of programmed death ligand 1 (PD-L1) expression in macrophages, which in turn inhibits T-cell function. The authors of this study propose that the underlying mechanism is the release of exosomes from ER-stressed HCC cells. These exosomes contain high levels of miR-23a-3p, which targets PTEN, thereby regulating PI3K/AKT signaling and PD-L1 expression [130]. Therefore, inhibiting miR-23a-3p could have therapeutic effects. Similarly, ER stress-induced exosomal miR-27a-3p derived from breast cancer cells indirectly inhibits PD-L1 expression in macrophages, thus promoting immune evasion [131].

Furthermore, the number of infiltrating lymphocytes is indicative of efficiency of anti-tumor immunity, which is modified by miRs. Zarogoulidis et al. showed that miR-155 activates the immune system and promotes tumor-infiltrating lymphocyte infiltration [132]. In addition, the combinatorial therapy of miR-155, blocking autophagy, is beneficial to increase chemo-sensitivity to carboplatin in lung cancer, thereby posing an option for therapeutic exploitation [132]. Moreover, miR-142-5p treatment enhances anti-tumor immunity by blocking the PD-L1/PD-1 interaction in pancreatic cancer: miR-142-5p overexpression in tumor cells decreases PD-L1 expression, subsequently increasing IFN-γ and TNF-α levels as well as infiltrating CD4^+^ T lymphocytes and CD8^+^ T lymphocytes, while decreasing PD-1^+^ T lymphocytes in vivo [133]. Furthermore, miR-183 targets PD-L1 and another immune checkpoint CTLA-4. Wei et al. demonstrated that miR-183 is a potential candidate for immunotherapy, as it showed anti-glioma efficacy by modulating the immune system: miR-183 overexpression in human CD4^+^ T cells lead to decreased CTLA-4, PD-1, and Forkhead box protein 3 (FoxP3) expression and in vivo miR-183 treatment of GL261 gliomas in immune-competent mice showed tumor regression, which could not be observed in immune-incompetent mice or after CD4^+^ or CD8^+^ T cell depletion. Furthermore, in vitro treatment of glioma cells with miR-183 at physiological levels had no suppressive effect, supporting its immunomodulatory function [134]. Regulatory T-cell-mediated immune suppression can also be mediated by tumor-secreted miR-214. MiR-214 targets PTEN and promotes Treg expansion and subsequent enhanced tumor growth [135].

The DC- or NK cell-mediated anti-tumor immune response can also be repressed by miRs. Exemplarily, miR-203-containing exosomes from pancreatic cancer cells suppress anti-tumor immunity. In recipient DCs, miR-203 inhibits the expression of TLR4, thus downregulating the production of TNF-α and IL-12 [136]. For NK-cells, Berchem et al. demonstrated that NK cytotoxicity and function is modulated by tumor-derived exosomal miR-23a and TGF-β via decreasing the expression of NKG2D activator surface receptors [137]. Another candidate for immunotherapy is miR-128, as it modulates the activity of several TME immune cells at once. miR-128 was found to inhibit pancreatic ductal adenocarcinoma (PDAC) growth and metastasis in in vivo experiments. As part of the underlying mechanism, the authors of the study propose enhanced anti-tumor immunity of DCs, CD8^+^ T cells and natural killer T cells (NKT). miR-128 regulates ZEB1 and inhibits CD47, thereby interfering with CD47-mediated immune evasion [138]. Likewise, miR-130a and miR-145 overexpression qualifies as a possible therapeutic approach targeted at the metastatic microenvironment and host anti-tumor immunity. Ishii et al. demonstrated that miR-130a and miR-145 are downregulated in Gr-1^+^CD11b^+^ immature myeloid cells. Ectopic miR-130a and miR-145 expression reprogramed the tumor-associated myeloid cells as well as skewed the microenvironment towards anti-tumoral [139].

Furthermore, Sasaki et al. proposed the genetical engineering of miR-17-92 expressing T cells as a promising approach for cancer immunotherapy. They found that the miR-17-92 cluster is downregulated in glioma patient samples as well as in murine Th2 cells in murine models, where the miR-17-92 downregulation could be reversed by disruption of IL-4 signaling. Additionally, miR-17-92 transgenic mice showed a superior type-1 phenotype in CD4^+^ T cells in comparison to wild type mice. Conclusively, miR-17-92 downregulation in T cells diminishes tumor control and the persistence of tumor-specific T cells [140]. Moreover, a study by Ledo et al. indicated that myeloid-derived suppressor cell (MDSC)-mediated immune suppression could be targeted by co-delivering miR-142-3p and the CCL2 chemokine. miR-124-3p-loaded nanocapsules reduced the immunosuppressive monocyte-macrophage subset and the CCL2 induced a potent monocyte-macrophage chemoattraction in in vitro studies in primary MDSC cultures [141]. Taken together, these studies demonstrate the potential for targeting miRs to enhance the immunotherapy efficiency.

### 4.2. MiR Therapeutics to Reverse Chemotherapy Resistance in the TME

Together with immune evasion, chemotherapy resistance is posing one of the big challenges in cancer therapy. This resistance can be mediated by various factors—one of them being miRs induced by TME *stimuli*, like hypoxia or cell–cell communication.

One characteristic feature of the TME is hypoxia due to insufficient vascularization of rapidly growing tumors. Hypoxia influences the miRome of cancer and stromal cells in the TME via downregulation of miR biogenesis machinery proteins or regulation of transcription factors that control miR expression. Consequently, several hypoxia-regulated miRs and their role in tumor progression have been identified. Some of the hypoxia-regulated miRs, e.g., miR-181b, miR-210, miR-26a, miR-424, miR-519c, and miR301-a, have also been associated with chemo- or radiotherapy response in different cancers [142,143,144,145]. Targeting these miRs for therapy could be a way to re-sensitize hypoxic tumors to therapies. For instance, in the hypoxic pancreatic cancer microenvironment, HIF-1α induces gemcitabine (GEM) resistance. Xin et al. showed that transfection of miR-519c, which is downregulated in pancreatic cancer, could inhibit HIF1-α in GEM-resistant pancreatic cancer cells under hypoxia. Therefore, a redox-sensitive nanoplatform co-delivering GEM and miR-159c was developed, which downregulates HIF-1α and genes responsible for glucose uptake and cancer cell metabolism, thereby significantly inhibiting orthotopic desmoplastic pancreatic cancer growth in NSG mice. Consequently, this treatment reversed hypoxia-induced chemotherapy resistance [146], showing the potential of miR therapeutics.

The crosstalk between tumor cells and stromal cells via miRs can also enhance chemotherapy resistance. It has been demonstrated that altering the miR transfer in the TME can be exploited for cancer treatment by a study showing that propofol prompts TAMs to secrete miR-142-3p, which conveys propofol action in cancer cells. It was first demonstrated that propofol inhibited tumor growth in tumor-bearing mice in an HCC model. Upon investigating the mechanism, it was shown that these effects were mediated by the delivery of miR-142-3p via secreted microvesicles from TAMs upon propofol stimulation [147]. Thus, interfering with miRs that are involved in TME-mediated therapy resistance is a possible way to reverse resistance and could be attempted in several scenarios: miR exchange between tumor cells and CAFs, TAMs, or other stromal or cancer cells or miRs involved in signaling that confers chemotherapy or immunotherapy resistance. Following, a few studies will be depicted that show the potential of developing therapeutic approaches, targeting miRs involved in the TME crosstalk-mediated drug resistance.

As an important cell type in the TME, CAFs are also involved in TME-mediated chemotherapy resistance via miRs. For example, cisplatin and paclitaxel promote CAF secretion of miR-522 in exosomes via activating the ubiquitin-specific protease 7 (USP7)/heterogeneous nuclear ribonucleoprotein A1 (hnRNPA1) axis. Exosome-derived miR-522 suppresses arachidonate lipoxygenase 15 (ALOX15) and decreases lipid-ROS accumulation in the recipient gastric tumor cells, which inhibits ferroptosis and decreases the sensitivity to chemotherapy [148]. In addition, miR-27a is transferred from fibroblasts to prostate cancer cells, where it increases resistance to chemotherapy by preventing p53 gene expression [149]. Zhang et al. found a correlation between resistance to gemcitabine and miR-21 expression in PDAC patients. They also demonstrated that miR-21 overexpression activated CAFs in vitro and promoted desmoplasia and increased gemcitabine resistance in PDAC, while downregulation had the opposing effect in vivo. Thus, they concluded that miR-21 regulated drug resistance in PDAC via CAFs [150].

The transfer of miRs from or to TAMs has also been shown to confer therapy resistance in several different studies. Challagundla et al. demonstrated that neuroblastoma cells polarize human monocytes to M2 macrophages via TLR8 activation by tumor-derived miR-21-containing exosomes. In turn, the TAMs secrete miR-155 in exosomes, which targets telomeric repeat binding factor 1 (TERF-1) in neuroblastoma cells, thereby increasing telomerase activity and subsequently cisplatin resistance [151]. In addition, TAM-derived exosomal miR-21 has been shown to confer cisplatin resistance in gastric cancer via inhibition of apoptosis and enhanced activation of PI3K/AKT signaling via PTEN downregulation [152]. Similarly, in epithelial ovarian cancer (EOC), hypoxic TAMs secrete miR-223, which enhances drug resistance in EOC cells via the PTEN-PI3K/AKT pathway [153]. In PDAC macrophage-derived exosomal miR-365 has been shown to weaken gemcitabine activation and to confer therapy resistance in vitro and in vivo. Molecularly, it is proposed to be upregulation of the triphospho-nucleotide pool in cancer cells as well as inactivation of gemcitabine via induction of the enzyme cytidine deaminase [154]. Upregulation of these miRs mediates chemotherapy-resistance, thus they are candidates for treatment with miR inhibitors. However, miR mimics could be equally valuable as shown by miR-770 in triple negative breast cancer (TNBC). miR-770 sensitizes TNBC cells to doxorubicin by downregulating STMN1 as shown by Li et al. [155]. They demonstrated that overexpression of miR-770 not only regulated apoptosis and EMT in cancer cells, but was also transferred to TAMs, where it affected macrophage polarization and antagonized M2 macrophage-induced chemotherapy-resistance.

Other stromal cells in the TME also influence therapy resistance. Bone marrow stromal cell-mediated therapy-resistance in AML is conferred via miR-23a-5p. Stromal cell-induced NF-kB signaling in leukemic cells downregulates miR-23-5p, which causes upregulation of protective autophagy via TLR2. Thus, leukemic cells are protected from chemotherapy-induced apoptosis [156]. Furthermore, it has been shown that doxorubicin treatment induced miR-21-5p expression in mesenchymal stem cells and derived exosomes. The exosome-derived miR-21-5p induced S100A6 expression in breast cancer cells, thus mediating chemoresistance in vitro and in vivo [157].

Finally, exosomal transfer of miRs can also happen between cancer cells, thereby conferring chemotherapy or radiotherapy resistance e.g., miR-155 has been shown to shuttle from resistant oral cancer cells to sensitive cancer cells, also desensitizing those cells to cisplatin [158]. Furthermore, exosome-derived miR-301a is involved in mediating glioblastoma radioresistance from hypoxic, resistant to normoxic, sensitive cells by targeting the tumor suppressor TCEAL7 gene, which negatively regulates Wnt/β-catenin signaling [159].

In most of the mentioned examples, a therapeutic approach would likely include overexpression or downregulation of the miRs to reverse chemotherapy resistance and be able to continue the therapy. However, as exosomal transfer facilitates miR-mediated chemotherapy resistance in some cases, disrupting this route of transfer could have a valuable impact on preventing resistance in the first place.

### 4.3. Co-Delivery

Several studies showed that delivering the miR drug together with a chemotherapeutic agent can improve the efficiency of the treatment. Polymer-based co-delivery systems have been reviewed by Dai et al. [160]. We describe some studies for the two most widely used miRs.

The most extensively used miR therapeutic in combinatorial delivery with chemotherapeutics is a miR-21 inhibitor. Simultaneous delivery of the cytostatic anticancer drug 5-FU and the miR-21 inhibitor oligonucleotide (miR-21i) to HER2-expressing cells in engineered exosomes demonstrated an anti-tumor effect in a colon cancer mouse model after systemic administration. MiR-21 target gene expression was rescued, thereby reducing tumor proliferation and induce apoptosis. Furthermore, treatment with the engineered exosomes reversed drug resistance and enhanced cytotoxicity in 5-FU-resistant colon cancer cells [161]. In gastric cancer, the same combination of 5-FU and miR-21 inhibitor was delivered in trastuzumab-conjugated nanoparticles. This strategy increased trastuzumab targeting and antibody-dependent cellular cytotoxicity, while also enhancing sensitivity of HER2-expressing gastric cancer cells to trastuzumab and 5-FU in vitro and in vivo [162]. Furthermore, co-delivery of 5-FU and anti-sense miR-21 in PAMAM dendrimers improved cytotoxicity and decreased migratory abilities of glioblastoma cells in vitro [163]. Another cytostatic, docetaxel, has also been employed for miR-21 co-delivery in TNBC. In vitro experiments showed improved chemosensitivity of TNBC cells to docetaxel treatment after treatment with so-called chitosomes. Chitosomes are self-assembling core-shell supramolecular nanovectors carrying anti-miR-21 and docetaxel [164]. Another study co-delivered miR-21 inhibitor and the cytostatic gemcitabine to pancreatic cancer cells using polyethylene glycol-polyethylenimine-magnetic iron oxide nanoparticles targeted to CD44. Application of those nanoparticles resulted in downregulation of miR-21 followed by upregulation of PDCD4 and PTEN as well as EMT suppression. Additionally, proliferation was inhibited, and clonal formation, migration, invasion, and apoptosis were induced in vitro. The nanoparticles, in vivo, accumulated at the tumor site and potently inhibited tumor proliferation and metastasis. The synergistic anti-tumor effect suggested the nanocarriers as a promising anti-cancer therapy in pancreatic cancer [165]. Furthermore, promising delivery vehicles for co-delivering miR-21 inhibitor and doxorubicin or epirubicin have been developed, some of which show synergistic anti-cancer effects of the therapeutics [166,167] or the ability to overcome multi drug resistance [168] and others, which show high delivery efficiency but no synergistic effect [169]. Similarly, a co-delivery system for an anti-miR-21 oligonucleotide and pemetrexed in cationic solid lipid nanoparticles for glioblastoma treatment shows promising uptake in vitro but no increased cytotoxicity [170].

As a sequential delivery of miR inhibitors and chemotherapeutic compounds can be critical for synergistic efficacy [171], Ren et al. designed nanoparticles to achieve sequential drug delivery. They employed a system of near-infrared-radiation (NIR)-responsive hollow gold nanoparticle (HGNPs) modified with PAMAM and loaded with miR-21 inhibitor and doxorubicin to target breast cancer cells in vitro and in a xenograft mouse model. Sequential delivery was achieved by first releasing the miR-21 inhibitor using the proton sponge effect of the PAMAM polymer after endocytic uptake of the nanoparticle. After 4h, NIR application collapsed the hollow gold-nanoparticles, freeing the encapsulated doxorubicin into the sensitized cancer cells. Anticancer efficacy increased 4-fold compared to doxorubicin only treatment after intravenous administration, showing the potential of this sequential delivery concept for cancer therapy [172].

The prominent tumor suppressor miR-34a is the second most used miR in combinatorial therapy approaches with chemotherapeutics as its downregulation is often involved in chemotherapy resistance. Thus, several approaches of co-delivery with cytostatics have been taken. Li et al. used polymeric hybrid micelles to deliver miR-34 and irinotecan in colorectal cancer cells. They showed enhanced anti-tumor effects due to the combined therapy in vitro and in vivo [173]. Another study by Shi et al. delivered miR-34 and paclitaxel in cationic solid lipid nanoparticles to melanoma lung metastases in mice where the nanoparticles showed potent synergistic anti-cancer efficacy [174]. Similarly, the delivery of miR-34a and docetaxel in nanocarriers inhibited tumor growth and metastasis in a metastatic breast cancer mouse model [175]. Delivering doxorubicin and miR-34a showed anti-tumor activity in prostate [176] and breast [177] cancer in vitro and in vivo. Furthermore, in uterine leiomyosarcoma, a maternal embryonic leucine zipper kinase MELK inhibitor (OTSSP167) may increase the sensitivity to doxorubicin via reversing MELK-induced M2 macrophage polarization via the miR-34a/JAK2/STAT3 pathway, subsequently promoting doxorubicin chemoresistance in the TME [178].

In addition to the combination with cytostatic drugs, miR-34a mimics have also been used in two further approaches. One study showed that co-delivery of miR-34a and sPD-1 in cationic lipid microbubbles (release via Ultrasound-targeted microbubble disruption (UTMD)) inhibited tumor growth and increased antitumor activity in cervical cancer in a xenograft mouse model [179]. Another study targeted miR-34a-carrying nanoparticles to Notch1-overexpressing TNBC cells using Notch1 antibodies. Here, the Notch1 antibodies had a dual role and did not only serve the purpose of being the targeting moiety, but also enabled suppression of Notch signaling. Additionally, the performed in vitro experiments showed regulation of miR-34a targets as well as induction of senescence and reduction of cell proliferation and migration [180]. Taken together, the developed systems for co-delivery show high potential to overcome chemotherapy resistance. Several other miRs that have been applied in co-delivery systems with chemotherapeutics are listed in Table 1.

## 5. Pharmacological Targeting of Pathways That Provoke Differential Expression of MiRs

Next to modulating miR expression via oligonucleotides, small molecules could also be employed. As those molecules do not need to interact with the miR itself (or substitute it), there are several stages in the miR pathway that can be targeted. One option is the pre-transcriptional targeting of miRs. To this end, promoters or their methylation status could be targeted. Additionally, interfering with the signaling cascades (starting from receptor activation) and transcription factors involved in miR expression could alter the miRome. Another way to influence miR expression is via targeting the biogenesis pathway. Describing all the involved pathways and targets for therapeutical interventions is beyond the scope of this review. We focus on the use of small compounds used to directly regulate miR expression in cancer.

However, an exemplary study demonstrating the implications that treatment with small compounds can have on the TME was performed by Chang et al., even if the miR is only regulated indirectly. They showed that miR-21 levels in the glioblastoma (GBM) microenvironment were associated with macrophage M2 polarization and temozolomide resistance. TAMs were secreting miR-21-containing exosomes, which increased tumorigenic properties and drug resistance in vitro. Furthermore, they propose a feedback loop of increased ability to promote M2 polarization by GBM cells with exogenously increased miR-21 levels via secretion of the M2 cytokines IL-6 and TGF-β1. Application of the STAT3-associated pathway inhibitor pacritinib reduced the release of miR-21-containing exosomes from TAMs as well as cell viability and colony formation associated with reduced levels of STAT3, Sox2, PDCD4, and miR-21 in GBM cells. Additionally, pacritinib application in a TMZ-resistant LN18-bearing mouse model showed its potential to overcome TMZ-resistance [205].

### 5.1. Upregulation of Tumor Suppressor MiRs

To influence global miR expression RNAi research aimed to identify small-molecule enhancers of microRNA (SMERs). Suppression of miRNA expression has been observed in cancer during normal expression of the miRNA biogenesis machinery components [206], as well as caused by downregulation of the miRNA-processing machinery components Drosha and Dicer [207,208]. Reduced Dicer and/or Drosha expression is associated with shorter survival in e.g., breast cancers [209,210,211]. Therefore, SMERs pose an attractive treatment option. The small molecule enoxacin, a fluoroquinolone antibiotic, has been shown to be an RNAi enhancer [212], that increases mature miR or siRNA levels. Furthermore, it reduces cell viability by enhancing maturation of downregulated tumor suppressor miRs in several different cancer models [213]. Furthermore, Chen et al. identified a universal activator of miRNAs from the photoreaction products of naphthalene-1,4-dione with acetylenes. The compound non-specifically upregulated endogenous mature miR levels by promoting pre-miRNA processing [214]. In combination with miR mimics, those mature miR enhancing agents could potentially add to the drug performance by increasing the amount of mature miR therapeutics.

In addition to the globally acting SMERs, small compounds for upregulation of specific miRs can also have therapeutic potential. This is shown, e.g., by rubone, a miR-34 activator that inhibits tumor growth in HCC in vitro and in vivo [215]. Furthermore, rubone has been used in combination with paclitaxel for micellar co-delivery to reverse chemoresistance in prostate cancer [216]. 

### 5.2. Downregulation of OncomiRs

Small molecules can also be employed for miR inhibition, but in this case targeting single miRs opposed to global miR expression. The so-called small molecule inhibitor of specific miRNAs (SMIR) approach aims to specifically decrease mature miR levels by targeting either the mature miR or any precursor form. One challenge that several studies face is the specificity for only the targeted miR, as numerous compounds efficiently decrease the targeted miR, but also others [217]. The first SMIR was found for miR-21. It decreased miR-21 levels by targeting the transcription of the miR-21 coding gene [218]. Later, several other SMIRS targeting miR-21 were identified [219,220,221,222,223]: Streptomycin was shown to bind the miR-21 precursor sequence, thereby interfering with Dicer processing and repressing miR-21 levels [220]. Naro et al. identified a small molecule inhibitor that perturbs miR-21 function, shows cytotoxicity and can reverse chemoresistance [219]. Additionally, topoisomerase inhibitors can bind the Dicer motif of oncogenic miR-21, thus inhibiting its processing both in vitro and in cultured cells. Target de-repression and inhibition of a miR-21-mediated invasive phenotype by the most potent compound could be observed [221]. There is also evidence suggesting that SMIRs can influence TME effects, as the miR-21 inhibiting compound AC1MMYR2 has been shown to impair CAF-induced metastasis in breast cancer [224]. These examples (more are listed in Table 2) show the potential of SMIRs as therapeutics for cancer treatment, and the ongoing research in this field is promising; also for several other miRs [225]. Several different methods for high-throughput screening of compounds or the computational design of SMIRs based on the target (pre-)-miR sequence have been developed, allowing for systematic discovery of new drug candidates [126,226,227,228,229]. Further examples for small compounds increasing miR levels are listed in Table 2.

## 6. Delivery of MiR Therapeutics

Delivering miR drugs poses challenges, from general considerations like the effect of miR mimics or antimiRs on endogenous miR expression, over oligonucleotide modifications to increase stability, to suitable delivery vesicles and targeting possibilities. For mimics especially, the saturable endogenous RNAi machinery is needed for them to function. For this reason, it is preferable to deliver miR mimics instead of precursor-miR oligonucleotides, to not influence the endogenous miR expression by saturating the miR biogenesis system [249]. However, there are pre-clinical studies successfully designing and evaluating bioengineered miR pro-drugs consisting of precursor-miRs that are coupled to tRNA in bacteria. This system has the advantage of not needing artificial RNA modifications and the pro-drugs’ potential for cancer therapy is supported by its capacity to influence target gene expression [250,251,252,253].

Administration of drugs can be performed systemically or directly into the tumor via injection. While intra-tumoral injection can enhance target specificity and efficacy, and minimize side effects [254,255], it is less useful for treating metastasizing tumors or leukemia [256]. Therefore, targeted approaches to systemic delivery are needed. In this section, we give an overview of the current state of the art. For additional details, we encourage you to read the reviews done by Rupaimoole et al. and Labatut et al. [257,258].

### 6.1. Oligonucleotide Modifications

Free dsRNA is degraded rapidly in plasma, so the stability and pharmacokinetics of the miR mimic or antimiR in the circulation need to be ensured. To this end, modifications can be made to the oligonucleotides. In general, phosphorothioate backbone modifications as well as 2′-alkylation (prevents RNaseH activation [259]) stabilizes the oligonucleotides, but are, especially for mimics, only tolerated up to a certain point as they hinder incorporation into the RNAi machinery [260].

For antimiRs different modifications have been tested, some of them being derived from siRNA technologies. As the functional blocking of the targeted miR is achieved by binding and no RISC incorporation is needed (opposed to miR mimics), antimiRs can be designed more freely. The use of a phosphorothioate backbone stabilizes antimiRs against degradation and increases the binding affinity to plasma proteins. Furthermore, 2′-O-methoxyethyl, 2′-O-methyl, or 2′-O-fluoro modifications and the use of locked-nucleic acids (LNA) have been shown to increase stability and/or binding affinity [95,257,261]. The LNA modification is the most successful modification and describes the methylene bridge between the 2ʹ-oxygen and 4ʹ-carbon in a ribonucleotide, which results in the nucleotide being locked in the C3′-endo conformation. This conformation favors pairing with RNA and increases specificity for the target sequence, while also improving endonuclease resistance [262]. So-called LNA mixmers [263] for miR-122 inhibition in the liver have been successfully tested in vivo in mice and non-human primates [264,265]. Those mixmers are consisting of two deoxyribonucleotides, followed by one locked ribonucleotide, to increase miR targeting efficacy. Furthermore, the passenger strand often has a higher amount of modifications and off-target effects can be reduced by the three-stranded nicked design, which ensures that no useable passenger strand exists [266].

### 6.2. Cellular Uptake

Even with increased stability in the circulation due to the chemical modifications, the delivery of miR drugs into the cells still poses a challenge as naked miRs are mainly taken up via endocytosis, eventually leading to miR degradation in late endosomes or lysosomes. The need for endocytic uptake results from the miR’s negative charge repelling the negative charge of phospholipids in the cell membranes. In addition, the lipophilic bilayer hinders the transition of the hydrophilic miRs through the cell membrane. However, one solution to achieve endosomal escape is the “proton sponge” effect, which is exploited by some polymer delivery strategies. Polymers containing unprotonated amines can absorb the protons that enter the endosome during its maturation to the lysosome. This proton absorption enhances an osmosis-induced influx of chloride anions and water, which results in the rupture of the endosome, liberating the miR drugs [267].

Another modification, designed to overcome the cell membrane barrier and improve tumor delivery, is the addition of a pH low insertion peptide (pHLIP) via a disulfide bond. The pHLIP undergoes a pH-dependent conformational change in the hypoxic/low-pH TME, which facilitates the formation of a transmembrane α-helix by the carboxyl terminus in the cell membrane. The cleavage of the disulfide bond in the cytosol subsequently results in the release of the cargo antimiR [268]. Similarly exploiting the unique chemical composition of the TME is an approach to engineer ROS sensitive polymers. Those polymers dissolve upon engaging ROS molecules in the TME, thereby releasing their miR drug cargo [269].

### 6.3. Delivery Vehicles for Oligonucleotides

One approach to increase stability and the possibility to target tumor cells is the encapsulation of oligonucleotides. Here, several different approaches can be taken, including viral vectors, polymer- or lipid-based vesicles, exosomes, or inorganic nanoparticles. Encoding the RNA molecules in adenoviral vectors showed some promising results in vitro [270] and in vivo [271,272]. However, viral vectors have some safety issues in the clinic due to immune reactions.

A widely used method is the packaging in polyplexes, consisting of polymers and complexed nucleic acids, which has been exploited in various studies [273,274,275,276]. Here, the toxicity needs to be balanced with the miR binding capacity. In general, cationic polymers have a higher miR binding capacity and interaction rates with cell membranes than neutral ones, but their charge is also associated with higher toxicity. The classically used cationic polymer poly(ethyleneimine) (PEI) enters the cell via endocytosis and uses the proton sponge effect for endosomal escape. It is employed in various delivery studies—sometimes used in combination with neutral polymers such as poly(ethylene-glycol) (PEG), to reduce toxicity. In line with PEI, cationic dendrimers, consisting poly(amidoamine) (PAMAM) or poly(propylenimine) (PPI) are delivering conjugated nucleic acids efficiently. Additionally, its comparatively low toxicity, biodegradability, and biocompatibility makes PAMAM preferable over PEI [27]. An even safer possibility are neutral Poly(lactide-co-glycolide) (PLGA) particles, which show no toxicity, but also a lower miR binding capacity. PLGA is also often combined with cationic peptides [268] or other polymers, like e.g., PEG, which improves encapsulation efficiency, circulation time, and bioavailability in animals [257,277,278,279]. A component being able to compromise between charge and toxicity, which could be well suited for miR delivery, could be the cationic polymer chitosan. It is derived from the naturally occurring chitin, a polysaccharide composed of glucosamine and N-acetylglucosamine residues. Chitosan shows low cellular toxicity, is biodegradable [258,280] and has been used for miR delivery in in vitro and in vivo studies [281,282,283]. Notably, chitosan nanoparticles without a targeting moiety have been used to deliver miR-33 to macrophages in mice to alleviate atherosclerotic lesions. The study showed that delivery of a functional miR mimic to macrophages is feasible [284]. As macrophage specificity could be enhanced by using targeting peptides, appropriately engineered chitosan nanoparticles could be employed in cancer treatment, targeting TAMs. Another study by Deng et al. used mannose-modified trimethyl chitosan [MTC]-conjugated nanoparticles carrying a miR-146b mimic for an immunotherapeutic approach targeted at intestinal macrophages in the DSS mouse model. They showed that M1 macrophage activation could be inhibited and concluded that the miR-146b nanoparticles could be used in immunotherapies for ulcerative colitis and colitis-associated cancer [285].

There are also a couple of lipid-based delivery methods like lipoplexes, stable nucleic acid lipid particles (SNALPs), or neutral lipid emulsions (NLEs). Lipoplexes are liposomes composed of a mix of lipids with cationic head groups and helper lipids, including e.g., some PEG chains, and the polyanionic nucleic acids [266]. Their overall positive charge facilitates binding to anionic cell surface molecules. The lipoplex composition can facilitate fusion with the cytoplasmic, endosomal, or nuclear membrane, depending on the characteristics of its components. As with the polyplexes, strategies e.g., for endosomal release can be incorporated: By adding a pH sensitive lipid head group, interaction with anionic phospholipids in the endosome can be facilitated, disrupting the endosomal membrane and releasing the RNA [262]. In addition, in line with polyplexes is the potential for toxicity that comes along with the cationic charge. Furthermore, it has been shown that lipoplexes show a higher toxicity in macrophages [286,287] and elicit pro-inflammatory responses [288]. Both effects could play a role in the TME and need consideration when designing miR therapeutics.

SNALPs are composed of cationic, fusogenic, and PEGylated lipids, can efficiently encapsulate nucleic acids and are therefore used in clinical programs for siRNA delivery [266] and have been used in in vitro [289] and in vivo studies for miR delivery in cancer models [290,291,292]. NLEs are composed of 1,2-dioleoyl-sn-glycero-3-phosphocholine (DOPC), squalene oil, polysorbate 20, and an antioxidant and only show low toxicity at the same time as showing a more equal distribution over the tissues and less liver accumulation as cationic lipoplexes [86,266]. Furthermore, DOPC-based nanoparticles for siRNA delivery have reached clinical trials and show promising results in preclinical studies for miR mimic delivery [89,293,294,295,296].

Inorganic nanoparticles have also been employed for miR transport in several studies. They are non-toxic, non-immunogenic, and are highly stable in vivo, but on the downside show non-specific binding affinities to functional groups in biological systems and colloidal stability [27]. The most extensively used inorganic vesicles for miR therapeutics are silica or gold nanoparticles (AuNPs). AuNPs have been shown to be able to deliver therapeutic miRs to tumor cells in models of breast [297] and prostate cancer [298] as well as hepatocellular carcinoma [299] and leukemia [300], amongst others. Similarly, silica nanoparticles have been used to deliver miR mimics to tumor cells—also in combination with chemotherapeutics. Silica nanoparticles dissolve upon hydrolysis, releasing their cargo, and need receptor targeting to be taken up into cells as they are not taken up by cells on their own [266,301]. If engineered accordingly, this may ensure cell type specificity, therefore potentially decreasing side effects, which are a huge challenge in cancer therapies.

Furthermore, in a study by Akao et al. ex vivo miR-transfected THP-1 macrophages shed miR-containing microvesicles after injection in mice. Based on their success they propose a treatment scheme where patient-derived macrophages could be transfected with therapeutic miRs ex vivo before re-injecting the macrophages for miR delivery [302]. Using exosomes as delivery vehicles solves the biocompatibility problem, but at the same time, a stable quality and composition is hard to guarantee.

### 6.4. Delivery Vehicles for Co-Delivery of Oligonucleotides and Other Pharmaceuticals

For encapsulation of different drugs their different physiochemical properties can also pose a challenge. MiR mimics or antimiRs are hydrophilic while chemotherapeutics, like paclitaxel, can be hydrophobic. Zhou et al. solved this issue by using calcium phosphate-polymer hybrid nanoparticles that could encapsulate both, the miR inhibitors as well as paclitaxel. Their strategy involved first encapsulating the oligonucleotides in calcium phosphate and then coating these capsules with an anionic lipid, dioleoylphosphatidic acid (DOPA), for co-encapsulation with paclitaxel inside the same nanoparticle. Another system, able to deliver a hydrophobic drug, doxorubicin (DOX), and a negatively charged miR, miR-34a, used reducible self-assembling polypeptide-based cationic micelles. The DOX is entrapped in the hydrophobic core of the poly(l-arginine)-poly(l-histidine)-stearoyl micelles, while the miR is bound to the hydrophilic outer shell. The study demonstrated that the system is able to simultaneously deliver DOX and miR-34a in vitro and in vivo. Furthermore, the micelles facilitate endosomal escape of miR34a as well as DOX release into the cell nucleus resulting in synergistic anti-tumor activity in prostate cancer cells [176]. Further examples for co-delivery systems of miR agents and chemotherapeutics are named in the section ‘Co-delivery’.

### 6.5. Tumor/TME-Targeting of the Delivery Vehicles

The vesicles, discussed above, achieve stability in the circulation and increase the half-life of the miR therapeutics. However, systemically administered miR drugs still face the challenge of reaching the tumor cells or their target cells in the TME. For this, different targeting strategies have been employed, including targeting via antibodies or stimulus-dependent drug-release. For instance, lipoplexes can be targeted to a certain cell type by using maleimide tethers as anchoring points for scFv ab fragments [303,304]. For miR-29b delivery to chronic lymphocytic leukemia cells, the oncofetal antigen receptor tyrosine kinase orphan receptor 1 (ROR1) was used for targeting the nanoparticles. Selectivity is ensured as only malignant B-CLL cells express ROR1 [305].

Another promising approach that is being tested in clinics is the targomiR approach. TargomiRs consist of a double-stranded, synthetic RNA molecule, non-living bacterial minicells (EDV^TM^ nanocells from EnGeneIC Ltd.) [306] as drug delivery vehicle and a targeting moiety like e.g., an anti-EGFR antibody [84]. In addition, targeting of miR-containing exosomes, that are generated e.g., by transfection of exosome producing HEK cells, can be achieved by transfection with the miR as well as a targeting moiety [307]. Another tumor-specific targeting moiety is hyaluronic acid (HA), which binds to CD44, a cell adhesion membrane glycoprotein that is overexpressed on metastatic breast cancer cells. Wang et al. used this to develop HA-coated PEI-PLGA nanoparticles that can potentially deliver chemotherapeutic agents and miRs for combinatorial cancer therapy [203].

Next to targeting the tumor via antibody or receptor-mediated approaches, general TME characteristics can be exploited to achieve tumor-targeting. The TME is unique in pH, temperature, redox potential, and levels of certain proteins/enzymes that can be targeted, and most solid tumors show a phenomenon called enhanced permeability and retention (EPR), which enhances lipid and macromolecular drug uptake in tumors. EPR is caused by the extensive angiogenesis and hyper-vasculature, defective vascular architecture as well as the impaired lymphatic drainage/recovery system, and a high abundance of a number of permeability mediators in the TME [308]. Additionally, the application of external stimuli, including light, ultra sound, or magnetism, to release drugs can be employed for targeted delivery of drugs to tumors [309].

One of the advantages of the modern possibilities of drug design is the high degree to which it is possible to customize the drugs. With sufficient engineering the encapsulation of the desired drug combination can be achieved and, as long as a specific targeting receptor can be found on the target cell, specificity can be increased, thus showing the high potential of this approach in cancer therapy.

## 7. MiR Therapeutics in Clinical Trials

Even though there is great potential in the clinical use of agents that modulate miR expression, there are no miR pharmaceuticals approved and no phase 3 studies listed on Clinicaltrials.gov to date. The highly related class of siRNA drugs is only slightly more successful on its way to clinical application, highlighting the number of challenges that need to be faced to successfully develop an oligonucleotide drug.

The difficulties of developing miR drugs for cancer treatment are also reflected in the clinical studies currently running. Clinicaltrials.gov lists several Phase 1 and 2 studies for miR agents aimed to treat different diseases, including diabetes, cardiovascular diseases, hepatitis C virus infection, and cancer with diverging outcomes. A prominent example of a miR drug that encountered problems in clinical trials is MRX34 from Mirna Therapeutics, Inc. The synthetic double stranded RNA oligonucleotide was administered by liposomal injection in patients with primary liver cancer to substitute depleted miR-34 and restore its activity on the p53/wnt cellular pathways. However, the study was terminated due to immune related serious adverse events and a planned phase 2 study in melanoma patients was withdrawn.

More promising results were obtained in other clinical studies examining drugs targeted at dysregulated miRs in cancer. Asbestos Diseases Research Foundation in cooperation with EnGeneIC Limited developed targeted minicells containing a miR mimic, so-called targomiRs. The first TargomiR, Mesomir 1, was tested in a phase 1 study in patients with malignant pleural mesothelioma. Mesomir 1 carries a miR mimic for miR-16, a tumor suppressor in several cancers. Targeting to EGFR-expressing lung cancer cells is achieved by an anti-EGFR bispecific antibody. According to the authors of the study, additional studies are supported by the acceptable safety profile and activity of the drug [84,310].

Another drug candidate aimed against a dysregulated miR in cancer was announced by Regulus. Their drug RGLS5579 aims to target miR-10b in glioblastoma multiforme, where it has been identified as a promising therapeutic target [311]. In their current study, they aim to confirm that miR-10b expression patterns in glioma samples can serve as prognostic and diagnostic markers and want to test the sensitivity of individual primary tumors to anti-miR-10b treatment in vitro. A positive study outcome would pave the way for their drug to enter clinical trials.

Furthermore, a few miR drugs in clinical trials for other pathologies are giving reason for hope if the same strategies can be applied to cancer treatment. That one class of miR drugs can work in different diseases is shown by locked nuclear acid (LNA)-based anti-miRs, which seem to be a promising class of miR drugs as human phase 1 studies of 3 LNA-based anti-miRs proved safety and activity of those agents. One is Miravirsen (SPC3649 from Roche), which targets the liver-specific miR-122 [312] and showed prolonged dose-dependent reductions in hepatitis C virus (HCV) RNA levels without evidence of viral resistance in a phase 2 study in patients with chronic HCV genotype 1 infection [313]. The second LNA-based anti-miR shown to be safe in humans is Cobomarsen (MRG-106), which targets miR-155 in multiple hematological malignancies [314]. Currently, there are two phase 2 clinical studies ongoing for the use of Cobomarsen in cutaneous T-cell lymphoma. The newest of the 3 anti-miR drug candidates is MRG-110, which inhibits miR-92a and is intended to promote angiogenesis and to be used in treatment of cardiac diseases. A phase 1 trial showed that MRG-110 reduces miR-92a levels and de-represses miR-92a target genes in the peripheral blood compartment. As target gene expression was especially altered in T cells and NK cells, the authors propose a potential therapeutic benefit in diseases with dysregulated immune functions [315], which could also become interesting in the tumor context.

Another promising approach to modulate miR expression in diseases is taken by Abivax. They are testing a compound, ABX464, in two phase 2 studies in subjects with Crohn’s disease or moderate to severe active ulcerative colitis, which often progresses to colon cancer. Administration of ABX4664 causes miR-124 overexpression by binding to the cap binding complex at the 5′-end of pre-mRNA and enhancing splicing of a single long noncoding RNA to generate the anti-inflammatory miR-124 [316].

Finally, UniQure Biopharma B.V. developed a gene silencing technology, using artificial micro-RNAs called miQURE™. For therapy of Huntington’s disease, they designed a AAV5 vector carrying one such miQure miR targeting the huntingtin gene (AMT-130), which is currently tested in a phase 1/2 study for evaluation of safety and proof of concept [317]. If the system proves to be safe and efficient in humans, it could probably be applied for cancer treatment as well.

## 8. Conclusions

Within the last decades, extensive studies of miRs in cancer have shown their impact on multiple cancerous gene and protein networks, suggesting them to be a hidden pattern behind malignant diseases. Understanding the regulatory pathways of miRs and their interactions, in both malignant cells and in tumor-infiltrating host cells, may be the prerequisite to take miRs one step further towards therapeutic application [318]. In vitro and in vivo studies have shown, that targeting a single miR or a target in a miR network is inefficient and cannot necessarily be transferred into the clinic. As mentioned before, miR-34 has been a promising target for treatment of liver cancer and liver metastasis. Unfortunately, administration of a miR-34 mimic had strong side effects in some patients. miRs, including miR-34, target several genes and in most cases not all interactions and molecules that can be affected by a specific miR are known, making it difficult to predict and to evaluate the side effects. In the case of miR-34, it can be speculated that the side effects might be caused by high concentrations of miR-34 in the blood or in the cell, thereby triggering undesirable toxic pathways of the miR agent. Thus, to proceed miR therapeutic agents into the clinic it is not enough to know the in vivo toxicity, but rather the entire regulatory network of a particular miR needs to be understood. This challenge is even more difficult since multiple miRs are not only dysregulated in malignant cells, but also in other cells of the TME, where they might have opposing functions. Therefore, it is inevitable to test the effect of a potential miR target in a comprehensive manner to assess their regulatory network in the TME and to gain insights regarding the context-dependent functions of the miR. The knowledge about the expression and function of immune-modulatory miRs is indispensable to fully elucidate the molecular mechanism underlying tumor progression and to predict the response and clinical outcomes of cancer immunotherapy. This knowledge would also be crucial in personalized medicine by precisely targeting a dysregulated signaling pathway in a particular cancer patient.

To date, there are some phase 1 and 2 clinical trials involving miR-targeting drugs, but no miR drug has entered a clinical phase III trial so far. This is partly due to the challenge of designing efficient miR carriers and delivery systems to specifically target cell types, tissues, and organs. The specific targeting of miRs to a cell of interest by using antibodies, ligands, and nanoparticles has been shown to enhance treatment efficacy and reduce off-target effects, including immunotoxicity [255], but there are still limitations. Using other miR carriers, for instance AGO proteins or low-density lipoproteins, in combination with a suitable delivery system might further increase the cellular uptake of miR therapeutics and their incorporation into RISC to enhance their regulatory functions. Developing strategies to directly target dysregulated miRs in TAMs or CAFs might be useful to restore anti-tumoral properties and enhance treatment responses. The application of miRs as cancer therapeutic molecules stands and falls with the efficacy and specificity of its delivery system and will be of relevance, once those miR carriers can progress from phase I/II to phase III. Since tumor progression is caused by the deregulation of multiple cellular pathways, traditional therapeutic approaches that target single proteins, as well as chemotherapy or radiotherapy alone are less effective. Combinatorial approaches with miR therapeutics have proven to reduce chemotherapy resistance and treatment efficacy in pre-clinical studies and, thus, pave the way for novel cancer therapies once the discussed obstacles have been overcome.

## Figures and Tables

**Table 1:** Pre-clinical studies for co-delivery of miRs and chemotherapeutics in cancer.

MiR Therapeutic	Chemotherapeutic	Delivery Vehicle	Cancer Type	Ref.
miR-7	Paclitaxel	PEG-PLGA-poly(l-lysine) nanoparticles	Ovarian cancer	[181]
miR-10 inhibitor	Paclitaxel	pH-sensitive liposomes	Breast cancer	[182]
miR-21 inhibitor	Doxorubicin	Core-shell tecto dendrimers	Breast cancer	[166]
		NIR-responsive hollow gold nanoparticles	Breast cancer	[172]
		Amphiphilic copolymers	Glioma	[169]
		Graphene oxide-based nanoparticles	Breast cancer	[168]
	Epirubicin	MUC1 aptamer-targeted poymers	Breast cancer	[167]
	Docetaxel	Chitosome	TNBC	[164]
	Cisplatin	Nano-Graphene oxide nanoparticles	Lung cancer	[183]
	5-FU	Engineered exosomes	Colon cancer	[161]
	5-FU	Poly(amidoamine) dendrimers	Human glioma	[163]
	Gemcitabine	Dendrimer-entrapped gold particles using UTMD	Pancreatic cancer	[184]
		PEG-PEI magnetic iron oxide nanoparticles	Pancreatic cancer	[165]
	Pemetrexed	Lipid-polymer hybrid nanoplexes	Glioblastoma	[185]
		Cationic lipid nanoparticles	Glioblastoma	[170]
	Sorafenib	Dual targeting reconstituted HDL	Hepatocellular carcinoma	[186]
	4-Hydroxy-tamoxifen	PLGA-b-PEG nanoparticles	Breast cancer	[187]
miR-29b	Retinoic acid	Micellar nano system	NSCLC	[188]
miR-31	Doxorubicin	Stimuli-responsive silica nanoparticles	HeLa cell line/tumors	[189]
miR-34a	Doxorubicin	Hyaluronic acid chitosan nanoparticles	TNBC	[177]
		Cationic polypeptide-based micelles	Prostate cancer	[176]
	Paclitaxel	Lipid nanoparticles	Melanoma lung metastases	[174]
	Irinotecan	Polymeric hybrid micelles	Colorectal cancer	[173]
	Notch1 antibody	PLGA nanoparticles	TNBC	[180]
tRNA-mir-34a	Doxorubicin	Creatin-based polymer	Breast cancer lung metastases	[190]
miR-34a activator rubone	Docetaxel	Dual responsive micelles	Prostate cancer	[191]
miR-122	Doxorubicin	Gold nanocages	Hepatocellular carcinoma	[192]
	5-FU	Chitosan nanoparticles	Hepatocellular carcinoma	[193]
miR-181a	Melphalan	Lipid nanoparticles	Retinoblastoma	[194]
miR-200	Irinotecan	pH-sensitive and peptide-modified liposomes and solid lipid nanoparticles	Colorectal cancer	[195]
miR-200c	Docetaxel	Gelatinases-responsive nanoparticles	Gastric cancer	[196]
miR-205	Gemcitabine	Cationic copolymers	Pancreatic cancer	[197]
miR-218	Temozolomide	Folate-chitosan gel-delivered gold nanoparticles	Glioblastoma & lung cancer	[198]
miR-221/222 inhibitors	Paclitaxel	Calcium phosphate-polymer hybrid nanoparticles	TNBC	[199]
miR-345	Gemcitabine	Polymeric dual delivery nanoscale device	Pancreatic cancer	[200]
miR-375	Cisplatin	Lipid-coated nanoparticles	Hepatocellular carcinoma	[201]
miR-451	Doxorubicin	Coated calcium carbonate nanoparticles	Multidrug resistant bladder cancer	[202]
miR-542-3p mimic	Doxycycline	HA/PEI-PLGA nanoparticles	TNBC	[203]
miR-1284	Cisplatin	CD59 receptor-targeted liposomes	Cervical cancer	[204]

**Table 2:** Small molecule compounds mediating upregulation (↑) or downregulation (↓) of cancer-relevant miRs.

miR (Regulation)	Compound	Effect	Ref
miR-33b ↑	Curcumin analog EF24	Suppression of EMT and migratory potential of melanoma cells	[230]
miR-34 ↑	Rubone	Inhibition of tumor growth in HCC and reversal of chemoresistance in prostate cancer	[215,216]
miR-99a ↑	Diaporine A	Tumor suppression in NSCLC cells via miR-99a/mTOR pathway	[231]
miR-138 ↑	α-solanine	Restriction of cell migration and invasion and increase of chemosensitivity and radiosensitivity of lung cancer cells	[232]
miR-203 ↑	Curcumin	Decrease of proliferation and increase of apoptosis via Akt2 and Src in bladder cancer cells	[233]
miR-214 ↑	Sulforaphane	Inhibition of cancer stem cell-like properties and cisplatin resistance in NSCLC	[234]
miR-485 ↑	EGCG	Restraining of CSC-like characteristics in NSCLC	[225,235]
A panel of tumor suppressor miRs ↑	Diallyl trisulfide	Inhibition of proliferation of osteosarcoma	[236]
miR-21 ↓	4-benzoylamino-N-(prop-2-yn-1-yl)benzamide compound	Increase of apoptosis, retardation of proliferation, and upregulation of PDCD4 in HeLa and human glioblastoma cells	[237]
	Triptolide	Reduction of proliferation and increase of apoptosis	[238]
	Topoisomerase-inhibitor compound	Target de-repression and inhibition of the miR-21-mediated invasive phenotype in breast cancer cells	[221]
	oxadiazole inhibitors	Cytotoxicity in several cancer cell lines; re-sensitizes RCC cells to chemotherapy	[219]
	Honokiol	Suppression of proliferation and induction of apoptosis via regulation of the miR-21/PTEN/PI3K/AKT signaling pathway in human osteosarcoma cells	[239]
Pre-miR-21 ↓	AC1MMYR2	Target de-repression and inhibition of TME in cancer cells; impairs high dose paclitaxel-induced tumor metastasis, reprogramms CAFs	[224,240,241]
	Inforna-designed [228] small molecule	Impairment of lung metastasis of breast cancer in a mouse model	[242]
	Chem-CLIP-Frag-Map-designed fragment-based dimer	Selective reduction of mature miR-21 in vitro and in cells	[227]
	Butylcycloheptyl prodiginine	Target de-repression and inhibition of cellular proliferation in colorectal cancer cells	[243]
miR-96 ↓	Inforna-designed [228] Targaprimir-96	Decreases tumor burden in a mouse model of TNBC	[244]
Pre-miR-372 ↓	Neomycin-nucleobase-amino acid conjugates	Anti-proliferative activity toward gastric cancer cells	[228,245]
pre-miR-373 ↓	Neomycin-nucleobase-amino acid conjugates	Target de-repression and inhibition of proliferation in gastric adenocarcinoma cells	[228]
miR-424-3p ↓	Baicalein	Inhibition of cell growth and increase of cisplatin sensitivity of lung cancer cells via the PTEN/PI3K/Akt pathway	[246]
miR-515 ↓	Dimeric compound	sensitized HER2- breast cancer ccells to Herceptin	[247]
miR-544 ↓	Inforna-designed [228] small molecule	Selective induction of apoptosis under hypoxia and sensitization to 5-FU in TNBC cells in vitro and growth inhibition vivo.	[248]
